# Signals for change: A regional examination of rural clinical trial participant perspectives on values, health behaviors, and motivations for enrollment and retention in remote settings

**DOI:** 10.1017/cts.2025.47

**Published:** 2025-03-13

**Authors:** Elizabeth A. Johnson, Danika L. Comey, Bernadette McCrory

**Affiliations:** 1 Mark & Robyn Jones College of Nursing, Montana State University, Bozeman, MT, USA; 2 Mark and Robyn Jones College of Nursing at Montana State University, Bozeman, MT, USA; 3 Norm Asbjornson College of Engineering, Mechanical and Industrial Engineering, Bozeman, MT, USA

**Keywords:** Rural health, community engagement, ethnography, decentralized trial, clinical trial

## Abstract

There is a known disparity in clinical trial enrollment of rural-dwelling residents in the United States, largely due to financial constraints and travel burden. A big data study of an Intermountain West rural-serving healthcare system reported strong retention rates of historically underrepresented populations with adapted approaches. This exploratory qualitative descriptive study describes the lived experience and perceptions of eleven rural residents who participated or were interested in clinical trials from this healthcare system. Thematic analysis of interviews identified co-existing dualities between culture and traditional trial models, which suggest adapted designs are necessary to achieve opportunity equity in rural regions.

Clinical trials rely upon the enrollment of persons representative of the disease or disorder of interest to validate claims of drug or device safety and efficacy in real-world settings. The challenge, particularly in low-resource communities, is reflected in clinical trial data in the varying degrees of clinical monitoring and cultural patterns of health associated with regional healthcare systems or unaffiliated sites otherwise dominated by academic medical centers and high-resource healthcare systems [1]. Overrepresentation by metropolitan centers is largely attributable to the relative decreased engagement of trial sponsors with rural areas, given the higher likelihood of costly external resources required (e.g., external specialists for procedures and adapted supply chain logistics). Rural residents experience longer travel times than those residing in metropolitan areas, which may be prohibitive to necessary enrollment for clinical trials requiring frequent visits [2,3]. Marginalized communities such as American Indian, Alaska Native, and geographically isolated groups have historical hesitancy toward unfamiliar research groups and clinical trials [4]. The result of higher operational costs, lower population to enroll, clinical research mistrust, and travel constraints is a general lack of access to clinical trial opportunities, particularly industry-sponsored trials [5,6].

Access to clinical trials is an objective of Rural Healthy People 2030 [7] given the potential to improve quality of life and decrease morbidity and mortality rates with increased clinical patient monitoring during active trial involvement [8,9]. Federal advocacy includes research inclusivity for geographically disadvantaged groups through the Clinical Treatment Act passed in 2020 and the DEPICT Act passed in 2022 [10–12]. While rural participants are willing to participate in trials when asked by a trusted provider, this population participates at a reduced rate compared to metropolitan and micropolitan counterparts [3,4,6,8].

Most rural communities remain small in census, and the number of trial participants is smaller still. There is a unique value proposition of this region with higher retention and recruitment rates of American Indian and older adult participants than the national average [13]. As critical access hospitals and regional care facilities feel financial pressure to remain solvent during current funding headwinds, community provider awareness and advocacy of clinical trial inclusion as part of service line availability is a viable option to keep doors open and enhance patient care outcomes [14]. Trial sponsors require access to culturally diverse communities via these local potential research sites with affirmative engagement and responsiveness to the enmeshment of clinical and research in the continuum of patient care [13,14].

## Purpose

To better understand the experiences of rural-dwelling patient’s participation in clinical trial opportunities, a collaborative team of nursing and systems engineering researchers from a land grant University in partnership with a large regional healthcare system in the region sought to better understand the personalized the journey and lived experience of a rural trial participant or those interested in participating. These interviews informed a larger project of shared information exchange and clinical research integration in the Electronic Health Record of the healthcare system such that key clinic sites across a wide geographic area were able to communicate key research details, which may inform clinical care or trial opportunities.

## Method and sampling

Interviewees were recruited from a purposeful sampling of established active or former clinical trial adult participants from the large healthcare system serving the Intermountain West. A semi-structured interview guide was used with grand and mini-tour questions to facilitate exploration around literature-informed topics, such as the meaning of being a rural-dwelling resident, elements of support during clinical trial participation, the experience of having the opportunity to participate in a clinical trial, and the perception of the clinical trial and the research enterprise. Virtual and in-person interviews were recorded, transcribed, and interpreted from June 2023 to January 2024 using manual coding techniques and thematic analysis [15].

## Results

A total of 11 participants completed recorded interviews. The majority of participants (informants) identified as White/Caucasian Non-Hispanic, while 1 informant (9%) identified as American Indian/Alaska Native. The sample was closely distributed between males (4, 36%) and females (7, 64%). The informants identified their chronological age into three categories: 35–50 (2, 18%), 51–65 (2, 18%), and 66+ (7, 64%). Counties where informants reside represent mostly southern Montana, spanning from eastern to western communities. Two informants (18%) are actively involved in a clinical trial, and 8 (73%) had participated in at least one clinical trial in the past, whereas one respondent (9%) has not yet engaged in a clinical trial but was interested in doing so. Trial participation experience included oncology, diabetes, and rare disease programs. Results from participants can be found in Table 1.

**Table 1.** Thematic Findings of In-Depth Interviews

Thematic Grouping	Category	Definition	Number of Thematic Units	Exemplars
**Motivation to Participate**	*Finding Altruism in Opportunity*	Altruistic tendencies associated with enrollment and active or repeat clinical trial participation	50	*Being a part of the [trial], giving back, and helping our world handle cancer [P01]* *I just… I have nothing [else] to try [P04]* *I wanna be part of that good. It was selfish cause I was hoping I was gonna get the device, and it would help me personally. But, diabetes is so widespread, and if you could find something in addition to what’s known [about diabetes], that’s what motivated me [P09]*
	*Seeking Information*	Description of information exchange or research to expand knowledge base related to clinical trials or the specific trial of enrollment	25	*I just dove into a bunch of research [about the clinical trial] [P04]* *So basically, more education around clinical trials. In general, it’s a tough topic [P09]* *I guess to summarize, I felt like I got bits of information [about the clinical trial]. But, had I not had the level of knowledge I do as a nurse [they would have known less] [P10]*
	*Next Steps*	The stepwise or chronological passage of activities or procedures related to clinical trial participation	18	*You don’t really get to hear about what happened [after the end of the clinical trial]. So, I assume it’s [investigational product] is working [P07]* *I kind of felt pressured to start stuff [investigational product] right away [P10]* *When they told me they were stopping my trial, and I’d have to leave Dr. XXX, that was really hard for me [P12]*
	*Participant Advocacy*	The process of developing or engaging in self-advocacy while identifying external advocates	16	*You have to be an advocate… tell the doctor what you’re looking for, [but] not as far as diagnosing yourself and saying this is what I have [P06]* *I think [the doctor] thought that [the clinical trial] was really beneficial to me. I think that was the reason that she wanted to help me stay on [the clinical trial] [P12]*
**Perceived Barriers and Limitations to Participation**	*Emotional*	The emotional reactions experienced or reciprocated by others during trial participation and disease treatment/progression	42	*We drove home crying all the way [P04]* *In reality, I don’t feel well. I tell them [my family] I’m fine [P08]* *I felt like I literally got hit by a load of bricks, because now I’ve thought about my kids [P10]*
	*Financial*	Descriptions of trial-related financial compensation, burden, or impact	33	*They paid my mileage from my home [P02]* *I asked about that [using the trial device after concluding participation], and it was like $75 a month and I just can’t stomach that [P07]* *Compensation was ridiculous and way too low compared to time commitment [P09]*
	*Travel*	The patterns, behaviors, and habits associated with traveling from the participant’s residence or point of origin to the research site	20	*I said, “Where’s the next one [clinical trial]?” He [doctor] goes, “Salt Lake City.” [P06]* *There was good support in that. They paid for us to drive down and a place to stay [P09]* *I traveled to Billings once every two weeks [P11]*
	*Procedural*	The characteristics of lived experiences associated with completing trial-related activities, procedures, or tasks	12	*I was comfortable with going through that [procedure with research nurse], and she did a very good job. It took a half an hour [P02]* *They were flexible [with schedule]. And that was really nice [P07]* *Even if they have a minimum wage job, I mean, my gosh, people are stepping away from their work, their livelihood, to do this [P09]*
	*Technical*	The influence of technology in clinical trial conduct or participation in a clinical trial	11	*I have a smart watch and what I did was set up an alarm when I want it to go off, so I would take the medication and then I would check the little box on my watch [P05]* *The struggle is real sometimes with technology [P06]* *With my kids here too, they keep me updated with the technology stuff [P11]*
**Facilitators to Participation**	*Safety*	Perception of safety related to the trial involvement (such as procedures, treatment, or devices) and clinical care integration with research activities	11	*However, [if it was] you know, life or death? Yes, I would go there [to the research site] [P06]* *They hear trial and they think, oh, you’re automatically gonna give a really scary drug to me or my kid, and I don’t want to take the risk. Where, if they understood [more about clinical trials], you know, maybe they’re in a phase three trial and you know what it’s gone through to get to that point [P09]* *I felt totally safe [P11]*
	*Awareness of Clinical Trial Opportunities*	Modalities identified as creating awareness to clinical trial opportunities	14	*I think I got an email about it [P05]* *Just kind of in passing [P07]* *That [access to novel medication] was the main reason that I wanted to try it [P11]*
	*Perception of Research Site*	Perceptions associated with the clinical research site that managed the clinical trial	9	*I did get my own copy [of the consent form], which was nice [P07]* *The mail is extremely convenient [P09]* *When I was in Billings, I had the same nurse, my trial nurse, the whole time. She was there, and so I had that constant [P12]*
**Rurality’s Influence in Clinical Trial Care**	*Trust in Others*	The personal connections and trust that participants place in the clinical trial team, the clinical care provider team, and the medical teams	29	*I like that she [research nurse] did that [verbal review of study] instead of just planting it all in front of me on paper and having us make a decision right then [P05]* *The reason is, if you’re not comfortable with your doctor or healthcare provider, you have a tendency to not share everything [P06]* *Telehealth is much more convenient, but much less personal [P08]*
	*Community Context*	Descriptions of a participant’s community resources, capacity for clinical trial support, and characteristics that influence the ability to enroll/remain on a trial	20	*It takes me an hour to get to work…and I worked 12-hour shifts. So, that decreases the amount of time in the week I’m able to get in [for the clinical trial] on a daily basis [P05]* *The reason why we’re interested in that [trial] is obviously, you know, for a lot of Montana, it’s really hard to get to Billings [P07]* *We were going to try to get a [support] group going, but I think part of the problem is so many people who come from so far away… to say I’ll come for a once-a-month meeting that’s not going to involve a doctor’s appointment…they’re just not going to do it [P10]*
	*Caregiving and Support*	The resources necessary to experience support while participating in a clinical trial, including necessary caregiving support	12	*There’s always a person that I trust, that I can talk to at [redacted support community organization]. I could get rides if I needed them…I just still am surrounded by support, good friends, and family [P01]* *We can take parental leaves through our insurance [P04]* *I know all those people, we take care of each other [P08]*

## Discussion

The results from these in-depth interviews present a depiction of rural clinical trial participant experience, motivation, and perceived barriers/facilitators to research engagement that align with other studies surrounding clinical trial equity and opportunity in underrepresented, geographically sparse populations. This work adds new insights into the complexities of rural community culture with engagement of caregivers and support persons, conceptually described as *networks* or *insiders,* that are the bedrock of supporting clinically congruent care once in their resident setting. While information surrounding clinical trial opportunities was important, it was the individual who was trusted by the informant that aided in the next steps and determined the overall experience of being on a clinical trial. These findings validate why other programs may not have been successful [16]. *Familiarity*, a rurality concept, can be reflected in a duality where it can inhibit motivation or can be supportive such as with physicians or research nurses as identified in this work who were trusted and seen as operating in the informant’s best interest [16].

A novel finding in this work was the perception of being rushed through the research process while grappling with a diagnosis, feeling the need to start activities “*right away* [P10]” however not hearing about the results of the clinical trial or how the individual participant contributed to the advancement of scientific knowledge related to the drug or device. The respondents’ emphasis on education about clinical research and the immediacy of trial procedures, such as informed consent, reflect the rurality concept of *resources,* where a rural participant appraises on-hand knowledge or material resource versus those absent but necessary to be successful toward a goal or action [17]. The concept of resources also reflects the knowledge created by the clinical trial as beneficial to not only society at large but also the immediate community. *Distance* is another concept that encapsulates the behavioral or perceived separation between two or more entities, which was described in this sample not only in the physical sense of travel for trial procedures but the separation of a participant from results and ascribed meaning to their individual journey on the clinical trial [17]. Together, *resources* and *distance* demonstrate the dual pressures placed on the participant and the community to learn at an expedient pace about the clinical research enterprise, the individual trial, and then appraise which resources are required to be in place to participate.

There was heightened discussion related to *isolation* and how this was combated through caregivers, support systems, or when these were notably absent [17]. *Professional isolation* was experienced by those who continued to be employed during the clinical trial and required more complex planning to support both work and research, such as working “*12-hour shifts* [P05]” and acknowledging the reduced earnings when a research visit was required if time was taken off work. Compensation provided by the clinical trial was not to an anticipated value beyond travel mileage [P02], with informants describing it hard to “*stomach* [P07]” the low compensation related to the *“time commitment* [P09].” More common was the leaning-in of caregivers, family, and spouses to be the additional knowledge repository, advocate, and cheerleader. The community is associated with a relational knowledge that can ameliorate isolation, with many including their healthcare providers and research team in this relational circle of support. There was acknowledgement among informants that shared communication was the bedrock of decreasing isolation and therefore safety and satisfaction in the experience of being a trial participant in a rural setting [P02, P06]. Extrapolation of results may be difficult due to the small sample size and unique nature of the population.

### Implications and forward momentum for reflecting rural culture in clinical trials

In alignment with informant reflections related to compensation congruent with participation time and effort, there is current legislative activity to remove taxation from these trial payments. The bipartisan bill, H.R. 7418, the Harley Jacobsen Clinical Trial Participant Income Exemption Act, seeks to reduce the financial burden associated with trial participants and ultimately lessen obstacles for underrepresented individuals to engage with novel treatments [18,19]. For some populations, taxation and reporting trial compensation as income can dissuade participation entirely for fear of deportation [19]. Inconsistent and confusing language in the informed consent form describes trial payments or compensation as taxable income, lending to the perception that trial participants may be considered as independent contractors to the Internal Revenue Service [20]. As clinical trials and clinical care blend treatment and disease monitoring strategies, participants may be at risk of misconceptions associated with delineating clinical trial benefits when standard of care diagnostics or assessments may be applicable for research billing. Informants noted in this study that their clinical care visits commonly melded with research visits or procedures, encouraging institutional review of informed consent form language and billing/coding practices that support full transparency in financial elements of value added to participant care and individual responsibilities associated with reporting trial benefits or compensation. The Centers for Medicare and Medicaid Services outlines research-related billing/coding practices as do private insurance companies.

The lived experiences of these informants will contribute to steering research on important elements to include in the information exchange, such as provider notification alerts of potential trial opportunities and specific treatment guidance for acute care providers such as those in the urgent care and emergency care settings. Additional research will include the creation of an online community advisory forum to unite the region in clinical trial opportunity selection and protocol development to ensure alignment of resources, need, and healthcare system capacity to safely conduct clinical trials. This work presents the lived experiences of rural-dwelling adult residents in one region of the United States. As a small sample across a large unaffiliated healthcare system, these findings may not be generalizable to other settings of rurality or those identifying as clinical trial participants. However, the findings in this qualitative study were aligned with quantitative analyses of patterns of trial participant experience and behavior in the region, which facilitated meaning saturation and robust synergistic interpretation [13].

## Conclusion

The assimilation of clinical research into the complexity of rural living presents a penultimate challenge for the clinical research enterprise as regulatory pressure mounts to broaden belonging in clinical trial participation to otherwise under-engaged communities. As demonstrated by these interviews in the Intermountain West, representation is a core belief and part of one’s purpose in the value of coming together for the greater good of medicinal progress. The resiliency of rural residents has been reflected in their own words and rings true to guide next steps for trial sponsors to rethink documented barriers and facilitators in a personalized vantage point. While dualities remain, such as telehealth feeling impersonal but the travel challenges to get to the research site, these are largely aspects of known rurality concepts that culturally have co-existed. Through this work, these rurality concepts and lived experiences are intertwined and shed insight on how research teams may interact with new communities and those already willing to contribute to our collective understanding of how innovative medicinal products may improve quality of life on the frontier.
